# Zinc finger X-chromosomal protein promotes growth and tumorigenesis in human osteosarcoma cells

**DOI:** 10.12669/pjms.294.3498

**Published:** 2013

**Authors:** Rui Jiang, Zhong-li Gao, Mei Sun, Xing-yi Zhang, Jin-cheng Wang, Han Wu

**Affiliations:** 1 Rui Jiang, Department of Orthopedic Surgery, China-Japan Union Hospital of Jilin University, 126 Xiantai Street, Changchun 130033, PR China.; 2 Zhong-li Gao, Department of Orthopedic Surgery, China-Japan Union Hospital of Jilin University, 126 Xiantai Street, Changchun 130033, PR China.; 3 Mei Sun, Department of Pathology, The Second Hospital of Jilin University, 218 Ziqiang Street, Changchun 130041, PR China.; 4 Xing-yi Zhang, Department of Thoracic Surgery, Department of Orthopedic Surgery, China-Japan Union Hospital of Jilin University, 126 Xiantai Street, Changchun 130033, PR China.; 5 Jin-chengWang, Department of Orthopedic Surgery, China-Japan Union Hospital of Jilin University, 126 Xiantai Street, Changchun 130033, PR China.; 6 Han Wu**,** Department of Orthopedic Surgery, China-Japan Union Hospital of Jilin University, 126 Xiantai Street, Changchun 130033, PR China.

**Keywords:** ZFX, siRNA, Osteosarcoma, Tumorigenesis

## Abstract

***Objective:*** To investigate the role of Zinc finger X-chromosomal protein (ZFX) in oncogenesis of Osteosarcoma tumor.

***Methods:*** Here, we first conducted an expression analysis of ZFX in Osteosarcoma cell lines. Then, we constructed ZFX-specific small interfering RNA (siRNA)-lentiviral vector that is capable of effectively inhibiting the expression of ZFX gene in human Osteosarcoma Saos-2 cells, and investigated systemically the impacts of ZFX silence on the growth and invasive ability of the cancer cells in vitro. Furthermore, we determined the effects of ZFX knockdown on the cell cycle distribution and apoptosis of Saos-2 cells.

***Results: ***We found that ZFX inhibition resulted in significantly impaired proliferation and colony formation as well as mitigated invasiveness of Saos-2 cells. Importantly, si-ZFX infected cells exhibited a greater portion of cells at G1 phase, but a minor portion of S and G2/M phase cells. Moreover, a greater portion of sub-G1 apoptotic cells was observed in si-ZFX infected cells.

***Conclusions: ***These results strongly suggest that ZFX is a novel proliferation regulator that promotes growth of Osteosarcoma cells, and downregulation of ZFX expression induces growth suppression of Saos-2 cells via arrested G0/G1 phase cell cycle and apoptosis pathways, thereby indicating that ZFX may serve as a new molecular target for Osteosarcoma tumor therapy.

## INTRODUCTION

Osteosarcoma is an aggressive malignant tumor arising from primitive transformed cells of mesenchymal origin. Despite a recent improvement in long-term survival rates, the prognosis of Osteosarcoma is still poor.^1^, Therefore, it is necessary to find new therapeutic molecular targets and strategies for the prevention and treatment of Osteosarcoma. It has been established that Osteosarcoma arises as a consequence of the accumulation of multiple genetic alterations involving critical genes that control cell proliferation and mitosis Patients with Osteosarcoma may find that alternative treatment methods are the best option. Many forms of treatment are currently being researched as more is learned about how cancer interacts with the body. Better understanding of genetic carcinogenic effect has led to a new era of targeted therapy in the management of Osteosarcoma.

Zinc finger X-chromosomal protein (ZFX) gene which locates on the X chromosome is structurally similar to a related gene on the Y chromosome. ZFX and Zinc finger Y-chromosomal protein (ZFY) have been applied in gender identification for several species. These genes encode a member of the krueppel C2H2-type zinc-finger protein family. The full-length ZFX protein contains an acidic transcriptional activation domain (AD), a nuclear localization sequence (NLS) and a DNA binding domain (DBD) consisting of 13 C2H2-type zinc fingers. Studies in mouse embryonic and adult hematopoietic stem cells showed that ZFX was required as a transcriptional regulator for self-renewal of both stem cell types. For years, the role of ZFX in human cancer remains unknown. Previous studies have demonstrated that that ZFX had an important function in the tumorigenesis of human laryngeal squamous cell carcinoma (LSCC). However, to date, the functional roles of ZFX in Osteosarcoma have not been demonstrated.

Small interfering RNA (siRNA) is a class of. siRNA plays a critical role in the (RNAi) pathway, where it interferes with the of specific genes with complementary nucleotide sequence.^,^ Applications of RNAi induction using siRNAs for mammalian cells have been well recognized. In this study, we adopted a lentiviral vector-mediated RNAi system to achieve highly stable silence of ZFX.

Here, we first conducted an expression analysis of ZFX in Osteosarcoma cell lines. Then, we constructed ZFX-specific small interfering RNA (siRNA)-lentiviral vector that is capable of effectively inhibiting the expression of ZFX gene in human Osteosarcoma Saos-2 cells, and investigated systemically the impacts of ZFX silence on the growth and invasive ability of the cancer cells in vitro. Furthermore, we determined the effects of ZFX knockdown on the cell cycle distribution and apoptosis of Saos-2 cells. As a result, we found that ZFX is a novel regulator, which modulates the proliferation and migration capabilities of Osteosarcoma cells via arrested G0/G1 phase cell cycle arrest and apoptosis.

## METHODS

***Cell culture: ***Human Osteosarcoma Saos-2 cell line (Cell Bank of Chinese Academy of Sciences, Shanghai, China) was routinely maintained in DMEM (Hyclone, Logan, Utah, USA) with 10% FBS and (Hyclone, Logan, Utah, USA) and penicillin/streptomycin at 37℃ at 37°C in a humidified atmosphere of 5% CO_2_ and 95% O_2_.

***Construction of ZFX siRNA with lentivirus packaging: ***To overcome drawbacks of short-time action and lack of regenerating ability of siRNA and permit robust inducible RNAi mediated ZFX silencing, the encoding short hairpin RNA (shRNA) lentiviral vector was constructed. The RNAi was designed based on ZFX (Gene ID: 7543) sequence (5’-GTCGGAAATTGATCCTTGTAA-3’) of oligonucleotides and negative control sequence (5’-TTCTCCGAACGTGTCACGT-3’) The sequences were annealed, and ligated into the Nhe I/Pac I (NEB, Ipswich, MA, USA) -linearized pFH1UGW vector (Shanghai Preii Co. LTD., Shanghai, China). The lentiviral-based shRNA-expressing vectors were confirmed by DNA sequencing.

***Letivirus infection: ***Recombinant lentiviral vectors and packaging vectors were then cotransfected into 293T cells using Lipofectamine 2000 (Invitrogen, Carlsbad, CA, USA), according to the instructions of manufacture for the generation of recombinant (lentiviruses ZFX shRNA (si-ZFX) and negative control shRNA (si-CTRL)). Supernatants containing lentiviruses expressing were harvested 72 hours after transfection. Lentiviruses were purified using ultracentrifugation. Saos-2 cells were infected with the lentiviruses at multiplicity of infection (MOI) =30. The uninfected Saos-2 cells were used as controls. 

***Quantitative Real-time PCR:*** Quantitative Real-time PCR was carried out using the method previously described by us.^,^ In brief, total RNA was extracted from Saos-2 cells 96 h after infection using the RNeasy Midi Kit (Promega, Madison, Wis, USA). Complementary DNA (cDNA) was synthesized with SuperScriptII reverse transcriptase (Invitrogen, Carlsbad, CA, USA). A mixture containing 1 μg of total RNA, 0.5 μg oligo-dT primer (Shanghai Sangon) and nuclease-free water in a total volume of 15 μl was heated at 70°C for 5 min and then cooled on ice for another 5 min. The mixture was supplemented with 2 μl 10×buffer and 200 U Super-Script II reverse transcriptase up to a final volume of 20 μl, followed by incubation at 42°C for 60 min; Real-time quantitative PCR analysis was performed using SYBR Green Master Mix Kit on DNA Engine Opticon TM System (MJ Research, Waltham, MA). Each PCR reaction mixture containing 10 μl of 2×SYBR GreenMaster Mix (TaKaRa, Dalian, China), 1 μl of sense and antisense primers (5 μmol/μl) and 1 μl of cDNA (10 ng), was run for 45 cycles with denaturation at 95°C for 15 s, annealing at 60°C for 30 s and extension at 72°C for 30 s in a total volume of 20 μl. For relative quantification, 2-ΔΔCt was calculated and used as an indication of the relative expression levels by subtracting CT values of the control gene from the CT values of ZFX. The primer sequences for PCR amplification of ZFX gene were 5′-GGCAGTCCACAGCAAGAAC-3′ and 5′-TTGGTATCCGAGAAAGTCAGAAG -3′. Glyceraldehyde 3-phosphate dehydrogenase (GAPDH) was applied as an internal control. The primer sequences of GAPDH were 5'-TGACTTCAACAGCGACACCCA-3' and 5′- TTGGTATCCGAGAAAGTCAGAAG.-3′. 

***Western blot: ***Western blot was performed using our previously described method with modifications.^, ^In brief, Saos-2 cell was collected and lysed by precooled lysis buffer after 96 h of infection. Total protein was extracted from cells and determined by BCA method. Twenty microgram protein was loaded onto a 12% SDS-PAGE. The gel was run at 30 mA for 2 h and transferred to polyvinylidene difluoride membrane (Millipore, USA). The resulting membrane was blocked in 5% non-fat dry milk blocking buffer and then probed with goat anti-ZFX (1:1000 dilution; Sigma, Cat# SAB4300451) and mouse anti-GAPDH (1:6,000; Santa Cruz, CA) overnight at 4°C. The protein level of GAPDH was used as control. The membrane was washed three times with Tris-buffered saline tween-20 (TBST), followed by incubation for 2 hours with anti-mouse IgG at a 1:5,000 dilution (Santa Cruz, CA). The membrane was developed using enhanced chemiluminescence (Amersham, UK). Bands on the developed films were quantified with an ImageQuant densitometric scanner (Molecular Dynamics).

***MTT assay: ***MTT (methylthiazol tetrazolium) assay was performed using the method previously described by us.^,^ Briefly, exponentially growing cells were inoculated into 96-well plates with 2×103 Saos-2 cells per well. After incubation for 24, 48, 72, 96 and 120 h, 10 µl of sterile MTT (5 mg/ml) was added into each well. Following incubation at 37°C for 4 h, the reaction was stopped by adding 100 µl of dimethyl sulfoxide. The formazan production was detected by measurement of the spectrometric absorbance at 595 nm. The values obtained are proportional to the amount of viable cells.

***Colony formation assay: ***Colony formation assay was performed using the method previously described by us.^,^ In brief, Saos-2 cells infected with si-ZFX or si-CTRL were respectively seeded in six-well plates (2×102 cells/well of Saos-2) and cultured at 37°C with 5% CO2 for 8 days. The cell colonies were washed twice with PBS, fixed in 4% paraformaldehyde for 15 min and stained with Giemsa for 30 min. Individual colonies with more than 50 cells were counted under a fluorescence microscope. 

***Fluorescence-activated cell sorting analysis: ***FACS flow cytometry analysis of cell cycle and apoptosis was performed using our previously described method.^,^ In brief, Saos-2 cells were seeded in six-well plates (1×106 cells/well of Saos-2). After 48 h, cells were collected, washed with PBS, and fixed with 70% cold ethanol. The cells were then incubated for more than 24 hours at 4°C. After washing the cells with PBS, propidium iodide (PI) was added to the cell suspension and the analysis of cell cycle distribution was performed by FACS can (Becton– Dickinson, Franklin Lakes, NJ, USA).

**Statistic analysis: **Data were expressed as mean ± SD. Student’s t test was performed to evaluate inter-group differences. P<0.05 was taken as showing statistical significance. All statistical analysis was performed with SPSS 10.0 software (SPSS10.0, Chicago, IL).

## RESULTS

**Fig.1:**
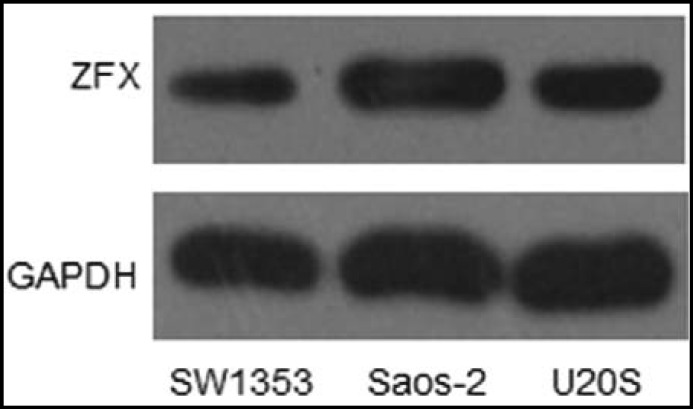
Expression of ZFX protein in different Osteosarcoma cell lines (SW1351, Saos-2, and U2OS cells).

***Expression of ZFX protein in different Osteosarcoma cells: ***To determine the expression levels of ZFX in different Osteosarcoma cell lines (SW1351, Saos-2, and U2OS cells), Western blot assay was performed. As shown in Fig.1, expression of ZFX was observed in the three different Osteosarcoma cell lines, however, the expression levels of ZFX protein in Saos-2 cells were significantly higher than that in SW1351 and U2OS cells. Therefore, Saos-2 cells were selected for RNAi-mediated gene silencing test.

**Fig.2:**
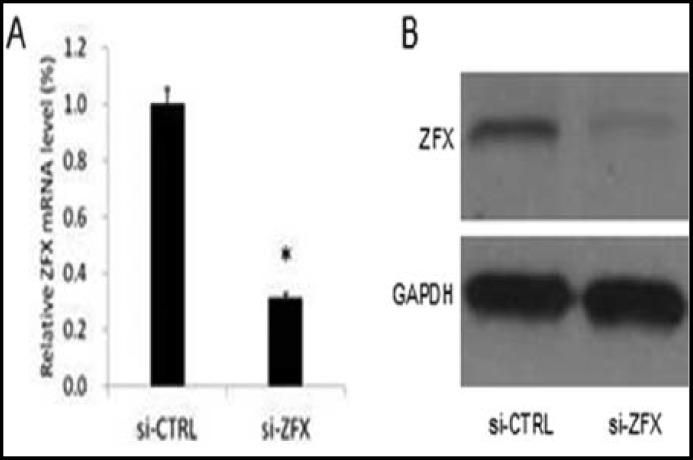
The mRNA and protein expression of ZFX in Saos-2 cells. (A) Real-time PCR result. (B) Western blot analysis. *P<0.05 versus si-CTRL

**Efficacy of lentivirus-mediated RNAi targeting ZFX: **To determine the silencing effect of lentivirus-mediated shRNA on ZFX expression in Saos-2 cells, Real-time PCR and Western blot analysis were performed after 120 h of infection. The mRNA expression of ZFX in Saos-2 cells infected with ZFX lentiviral shRNA was significantly decreased (Fig.2A). The expression of ZFX protein was significantly decreased after infection (Fig.2B). Therefore, the results indicated that the ZFX lentiviral shRNA vector exerted an obvious knockdown effect on targeted Saos-2 cells.

**Fig.3:**
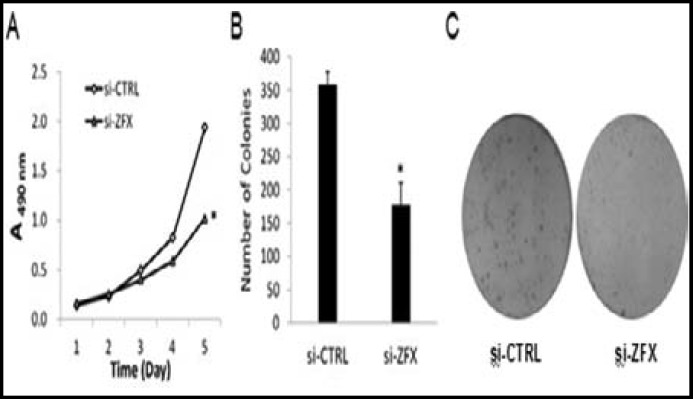
The proliferation of Saos-2 cells after si-ZFX infection. (A) MTT assay result. (B) Colony formation assay result. (C) Images of colonies recorded under microscope. *P<0.05versus si-CTRL

***Impact of downregulation of ZFX expression on cell growth in vitro: ***To explore the functional role of ZFX in the proliferation of Osteosarcoma cells, the growth dynamics of Saos-2 cells infected with si-CTRL and si-ZFX was determined by MTT assay and Colony formation assay, respectively. MTT assay showed that, during the 24 hours incubation period, the growth of si-CTRL infected cells did not differ from si-ZFX infected cells, whereas the growth of si-ZFX infected cells was prominently slower than si-CTRL infected cells at the time points of 72 hours, 96 hours and 120 h hours (Fig.3A). Quantitative analysis of colonies showed that after incubation for 8 days, the number of colonies in si-ZFX infected cells was lower than that in si-CTRL infected cells (P<0.05, Fig. 3B). Therefore, the low viability and colony-forming efficiency of si-ZFX infected Saos-2 cells demonstrated that downregulation of ZFX expression inhibits the growth of Osteosarcoma cells in vitro.

**Fig.4:**
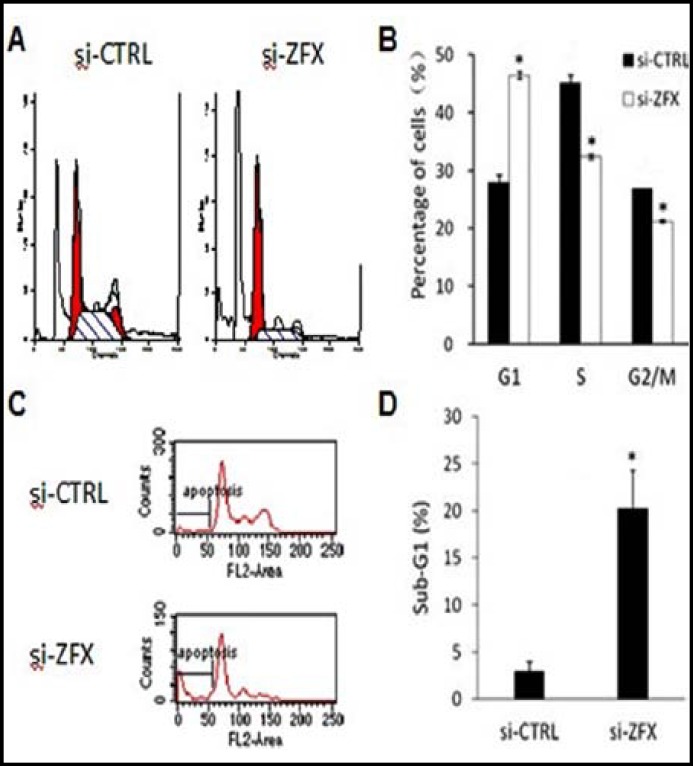
Effects of ZFX expression on cell cycle progression and apoptosis in Saos-2 cells. (A) Histograms of FACS analysis. (B) Cell cycle distribution (C) Images of FACS analyses at sub-G1 phase. (D) Percentage of sub-G1 phase cells. *P<0.05 versus si-CTRL

**Effects of knock-down of ZFX on cell cycle progression and apoptosis: **To determine the potential mechanism underlying the role of ZFX in the growth of Saos-2 cells, the cell cycle profile of si-CTRL and si-ZFX groups were determined by Fluorescence-activated cell sorting (FACS) analysis 96 hours after infection (Fig.4A). The frequency of infected cells at G1 stage was significantly higher in si-ZFX group than that in the si-CTRL group. In contrast, the frequency of infected cells at S and G2/M stage was lower in si-ZFX group than that in the controls (p<0.05, Fig.4B). As shown in Fig. 4C and D, there was an obvious difference in the cell population at sub-G1 phase between the two groups, suggesting that down regulation of ZFX expression might trigger apoptosis in Osteosarcoma cells. These results demonstrated that downregulation of ZFX expression resulted in cell cycle arrest and apoptosis.

## DISCUSSION

Osteosarcoma is an aggressive malignant tumor with multitude of cellular components and patterns of gene expression that affect tumor development.^,^ In-depth understanding of the molecular mechanisms underlying tumor proliferation is critical for the development of optimal therapeutic modalities. Moreover, there is an evidence to suggest that therapeutic drugs specifically targeting tumor-related molecules are expected to be highly specific to malignant cells and have minimal adverse effects due to their actions through well-defined mechanisms.^,^ During the ensuing decade, cohesin is emerging as the master regulator of genome stability and its related genes have been found to be highly relevant to diverse human malignancies.^,^ In the present study, we determined the expression levels of ZFX expression in Osteosarcoma Saos-2 cell line using quantitative Real-time PCR assay and Western blot analysis, and observed an obvious expression of ZFX in Osteosarcoma cells. Consequently, this led to a hypothesis that as an indispensible subunit of cohesin complex, ZFX may play a functional role in biological behavior of Osteosarcoma.

In this regard, we adopted a lentiviral vector-mediated RNAi system to further determine the roles of ZFX in the growth and invasive ability of Osteosarcoma cells. Using a constructed lentivirus expressing ZFX-specific siRNA, we infected Saos-2 cells to silence endogenous ZFX and investigated the impact of ZFX knockdown on the Osteosarcoma tumor development in vitro. We found that downregulation of ZFX expression greatly impaired the proliferation and colony-forming ability of Saos-2 cells evidenced by MTT assay. Importantly, we observed that ZFX knockdown caused cell cycle arrest at the G1 phase of the cell cycle, evidenced by the accumulation of G1 phase cells and decrease in S and G2/M phase. In addition, ZFX silence induced apoptosis as characterized by the prominent presence of sub-G1 apoptotic cancer cells. Collectively, these findings represent the first report that ZFX is a novel proliferation regulator in Osteosarcoma tumor.

ZFX, as a transcriptional regulator for certain tumors, is implicated as an important molecular target of malignancies. Our observation is consistent with the previous report and emphasized that ZFX facilitates important regulatory roles in Osteosarcoma cell proliferation and invasiveness. However, to date, the issue regarding whether and how ZFX interacts with other regulators is poorly understood, and further investigation is warranted to elucidate the detailed mechanisms underlying the action of ZFX.

## CONCLUSION

In conclusion, our findings strongly suggest the significance of gene ZFX in modulating the growth and invasiveness of Osteosarcoma tumor, and indicate that downregulation of ZFX expression induces growth suppression of Osteosarcoma Saos-2 cells via arrested G0/G1 phase cell cycle and apoptosis pathways. Hence, this study extends our knowledge about the oncogenesis of Osteosarcoma, and indicates that ZFX may serve as a new molecular target. 
